# Community voice in cross-sector alignment: concepts and strategies from a scoping review of the health collaboration literature

**DOI:** 10.1186/s12889-021-10741-9

**Published:** 2021-04-13

**Authors:** Aliza Petiwala, Daniel Lanford, Glenn Landers, Karen Minyard

**Affiliations:** Georgia Health Policy Center, Andrew Young School of Policy Studies, Georgia State University, 55 Park Place 8th Floor, Atlanta, GA 30303 USA

**Keywords:** Cross-sector alignment, Collaboration, Community, Health outcomes, Health equity, Health disparities

## Abstract

**Background:**

Health care access is an important driver of population health, and factors beyond health care also drive health outcomes. Recognizing the importance of the social determinants of health (SDOH), different actors in the health care, public health, and social service sectors are increasingly collaborating to improve health outcomes in communities. To support such collaboration, the Robert Wood Johnson Foundation developed a cross-sector alignment theory of change. According to the cross-sector alignment theory of change, community voice is critical for helping collaboratives address community health needs. Yet research on health collaboratives offers mixed guidance on how community voice should be understood and which community voice strategies are most effective.

**Methods:**

This study addresses a gap in the literature with a systematic scoping review of research on health-oriented cross-sector collaboration and community voice. By scanning key academic journals, searching three academic databases, and obtaining documents from across our professional networks, we identified 36 documents that address community voice in health collaboratives.

**Results:**

The review reveals several conceptions of community voice and a range of community voice strategies. We find that community voice strategies fall on a spectrum between two broad types of approaches: active and passive. These vary not only in the level of power shared between communities and collaborators, but also in the level of involvement required from the community, and this in turn has important implications for community collaboration strategies. We also find that while most strategies are discussed in the context of short-term collaboration, many also lend themselves to adoption in the context of sustainable collaboration and, ultimately, cross-sector alignment.

**Conclusion:**

This review provides a characterization and conceptualization of community voice in health-oriented collaborations that provides a new theoretical basis for future research. Passive and active community voice strategies can be studied in more detail for their expected impact on health outcomes and disparities. Increased attention to active community voice and the resources it requires can help practitioners achieve improved health outcomes and researchers understand the pathways to health improvement through collaboration.

## Background

Health care is important for improving population health and reducing health disparities. However, health outcomes are also driven by factors beyond health care, especially the social determinants of health (SDOH) such as socioeconomic status, living environment, and access to healthy food [1, 2]. Accordingly, efforts that link health care, public health, and social services are likely to improve population health and reduce health disparities. Research has demonstrated a link between cross-sector collaboration and health and health-related outcomes, including a reduction in deaths from cardiovascular disease, diabetes, and influenza [3]; improvements in children’s asthma control [4]; favorable trends in blood pressure control in patients with hypertension [5]; and increases in hepatitis B knowledge, testing, vaccination, and follow-up visits [6].

**Fig. 1:**
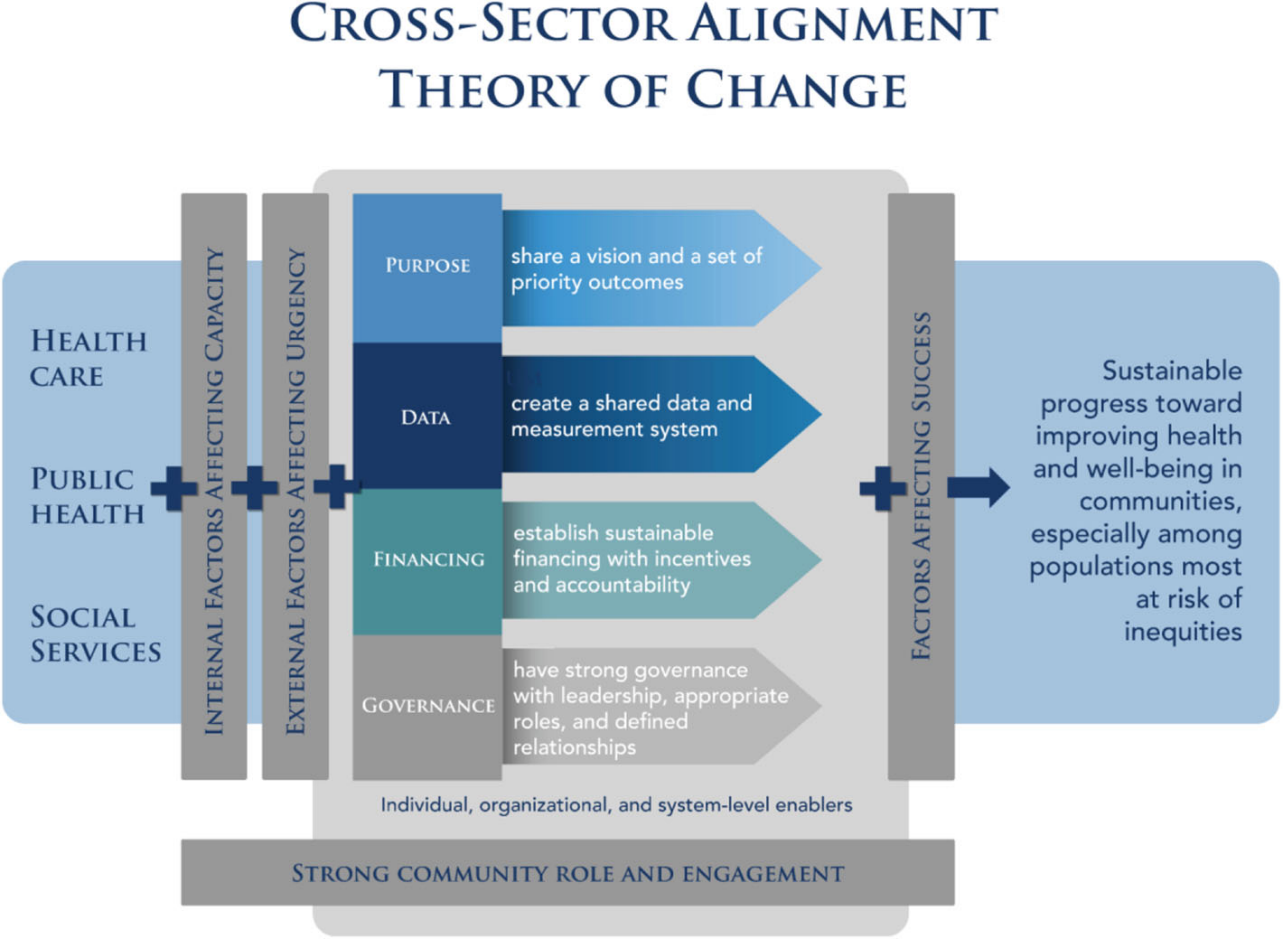
The Cross-Sector Alignment Theory of Change. This image was reprinted from Landers, G., Minyard, K., Lanford, D., & Heishman, H. (2020). A theory of change for aligning health care, public health, and social services in a time of COVID-19. American Journal of Public Health, 110(S2), S178-S180 [11]

While there have been many successes, a persistent problem is that health oriented cross-sector collaborative efforts are often fleeting, failing to establish sustained systems change that endures and continues to improve lives after initial energy wanes [7–10]. To address this problem, the Robert Wood Johnson Foundation (RWJF) recently drew on its many years of experience in the field of health-oriented collaboration, the growing body of research on cross-sector collaboration, and recent trends in practice around Collective Impact and Accountable Communities of Health to develop the cross-sector alignment theory of change (see Fig. 1). The central idea of the cross-sector alignment theory of change is that health care, public health, and social services might better meet the goals and needs of the people they serve over the long-term if they create collective change in four core areas: shared purpose, data, governance, and financing, so long as those changes reflect the will of the community in question [11].

Yet while research on health collaboratives has been steadily growing [12], recent reviews in this field have identified a need for increased attention to community voice. These reviews note that a key challenge in both practice and research is that there is little agreement in the literature on the definition of community voice or on the most important factors to consider when planning strategies for incorporating community voice in health collaboratives [12, 13]. Furthermore, many of the community voice strategies discussed are rooted in a paradigm that focuses on short-lived collaboration rather than sustainability and, ultimately, systems change [14]. In a time of greater recognition of the need for inclusiveness, it is especially important to understand the factors driving different community voice strategies and the implications these strategies have for improving population well-being over the long term. The purpose of this review is to build on early efforts to define community voice in the context of health collaboratives, identify specific community voice engagement strategies for health collaboratives along a spectrum of empowerment [12, 15, 16], and draw out the implications different community voice strategies have for sustainable cross-sector alignment.

## Methods

We conducted a scoping review for this study. Scoping reviews differ from Cochrane-style systematic reviews in that, while Cochrane-style reviews focus on an established and narrow literature, a well-defined question, and the weight of evidence bearing on that question, scoping reviews are optimal for synthesizing broad literatures, addressing broad questions, or laying foundations for future research in an emerging field [17]. We employ the scoping review method specifically to summarize and disseminate prior research findings on community voice in health-oriented cross-sector collaboratives, to draw out common themes, and to identify gaps that might be addressed in future research that subsequently helps shape practice [17].

### Data collection

Data collection involved two phases. In phase one, we identified papers addressing health-oriented cross-sector collaboration for a broader project on health-oriented cross-sector collaboration (661 papers) [authors, forthcoming]. In phase two, the set of papers was narrowed to those that also substantially addressed community voice (36 papers). Specifically, papers were read and manually excluded if community voice or a similar concept (e.g. community engagement, community agency, community participation, etc.) was not discussed or if community voice was identified as important but the paper did not elaborate on why it was important, discuss how it might come to be, assess what it might produce, or examine what it might entail (for a similar approach, see Calancie et al. 2021) [12].

Phase one consisted of three steps. First, papers were collected through a systematic scan of academic search engines. Second, we performed a systematic scan within journals commonly represented among the search engine results. For the third step, we conducted a purposive scan for relevant documents using general search engines, website searches, and professional networks.

The scan of academic search engines was conducted using Academic Search Complete, PubMed, and the Cochrane Library. Each search used the following search terms: (multisector OR multisector OR “multi sector” OR cross-sector OR “cross-sector” OR intersectoral OR inter-sectoral OR multisite OR multi-site) AND (collab* OR partner* OR integrat* OR joint OR alliance OR allied OR coalition) AND health AND (((healthcare OR “health care”) AND (social OR communit*)) OR ((healthcare OR “health care) AND “public health”) OR ((social or communit*) AND “public health)).

Documents were included in phase one if the following criteria were met:

- Published within the last 10 years
- English text version available
- Discussed at least two of the three sectors identified in the cross-sector alignment theory of change: health care, public health, and social services

All documents were independently reviewed for inclusion by two researchers. Disagreements on which articles to include or exclude were reconciled as a team.

The second step involved a systematic scan of key journals based on their frequency of appearance in the academic search engine results. Frequently represented journals included *Health and Social Care in the Community*, *International Journal of Integrated Care*, *Social Work in Public Health*, and the *Journal of Public Health Management and Practice*. Researchers used the same inclusion and exclusion criteria described above.

The third step involved a purposive scan for relevant research on health-oriented cross-sector collaboration. This involved gathering relevant papers forwarded by RWJF and other practitioners in the field, conducting a systematic scan of the RWJF website for relevant work, scanning for reports on websites of key organizations, collecting documents identified through RWJF’s and the authors’ professional contacts, and searching on general search engines using the search terms identified above.

In phase two of data collection, an additional inclusion criterion was added to the existing criteria:

- Addressed community voice or a similar concept (e.g. community engagement, community agency, community participation, etc.) and either elaborated on why it was important, discussed how it might come to be, assessed what it might produce, or examined what it might entail

**Fig. 2:**
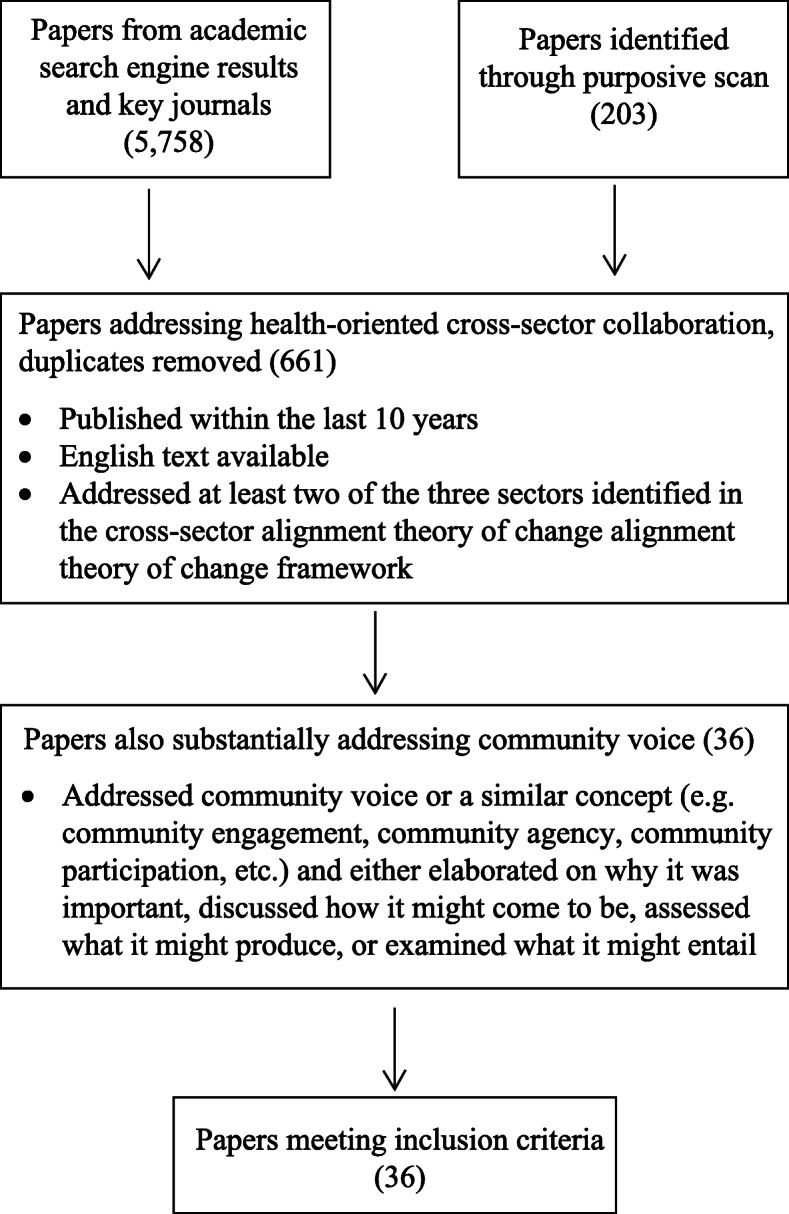
PRISMA Diagram

This criterion narrowed the set of papers to those specifically addressing community voice or a similar concept in the context of health collaboratives (36). Phase two also involved a directed review of research on community voice outside of health collaboratives which helped provide the conceptual grounding for this study and helped situate the findings within the broader literature (see Fig. 2).

### Coding

Information for each document in the review was coded in NVivo. Initial codes were based on a preliminary reading of the documents and included: “defining community voice”, “types of community voice”, “strategies for including community voice in collaboration”, “barriers to including community voice in collaboration”, and “notable passages”, with “community voice” here representing a broad class of similar concepts such as community participation, community agency, and community engagement. A first round of coding was manually completed using these initial codes. The coders then met to identify common themes, and a second round of coding was manually completed based around these themes. The results below reflect these themes and the subthemes that emerged during the second round of coding and are organized into three sections: conceptualizing community voice; types of community voice strategies; and sustained systems change and community voice.

## Results

### Conceptualizing community voice

Community voice is conceptualized in the health-oriented cross-sector collaboration literature in a variety of ways. The documents we reviewed used several terms including “community engagement”, “community agency”, “community participation”, and “consumer participation”. The papers we reviewed did not generally denote or connote differences in meanings between these terms. Accordingly, this study uses “community voice” as an all-encompassing term for inclusion of community members in collaborative activities.

Consistent with research on community involvement more broadly [18], many papers on community voice in health collaboratives do not formally define community voice terms. We found three exceptions, one from the Centers for Disease Control and Prevention (CDC), another from the National Academies of Sciences, Engineering, and Medicine (NASEM), and a third from Calancie and colleague’s recent review of health collaborative models. The CDC defines “community engagement” as “the process of working collaboratively with groups of people who are affiliated by geographic proximity, special interests, or similar situations with respect to issues affecting their wellbeing” [19]. NASEM defines “community agency” as “collective control, connections, capacities, and opportunities, including partnerships with shared decision-making and mutual accountability” [20]. Calancie et al. define “community engagement” as “working with community members and community organizations to build awareness around an issue, gain insight into an issue, and/or develop capacity within community members” [12].

Additional definitions are available in the broader literature and could readily be applied in future studies to help organize the learnings [21–25]. For example, Butterfoss’s definition of “community participation” is “a process along a continuum that enables communities to maximize their potential and progress from individual action to collective social and political change.”

There are potentially important differences between these definitions. For example, the CDC definition places emphasis on a shared commonality bringing communities together, while the NASEM definition emphasizes decision-making, accountability, and community agency. Calencie’s et al.’s definition emphasizes awareness and capacity building, and Butterfoss’s definition emphasizes the idea of a process, a spectrum, and the goal of socio-political change. Such differences have implications for the implementation of community voice strategies, for example by implicitly referring to different populations with different levels of power and capacity conveyed to the community in question. While we do not expect a single definition of community voice will be more effective than all others in every situation, this finding does underscore the need to explicate definitions when community voice terms are used in future practice and research.

While the papers we reviewed often used community voice terms interchangeably and did not always define those terms, we were able to identify several distinct dimensions along which community voice is generally discussed. These dimensions include: (1) the bounds used to define “community” versus “consumer”, (2) the population composing community voice, and (3) the depth of community voice strategies. These dimensions are discussed below.

#### Community and consumer voice

Community and consumer voice are generally used interchangeably. However, these terms have distinct implications, making it problematic to use them interchangeably. The term “consumer” tends to reference users of healthcare services [26–33] or those with lived experience [34]. The term “community” tends to reference groups of people in a bounded geographical location. Health care consumers and other community groups should be distinguished clearly and identified distinctly to promote conceptual clarity.

#### The population composing community voice

Community voice is provided by many different populations. Community voice could come from individuals living in one specific geographical location or users of specific services, as discussed above. Community-based organizations, themselves, may serve as proxies for community voice [35]. Because different populations can provide community voice, care must be taken to identify the specific population in question. For example, in some cases, an organization leader may be relied upon to speak for the community, while in other cases it is people being represented who are considered the voice of the community. These different categories of people have different experiences and backgrounds, and they may live in different communities. Accordingly, the meaning of community voice is likely to vary depending on the population providing that voice, and the population involved is likely to influence the systems changes promoted by a collaborative. This finding reflects the broader literature on community voice, which highlights the importance of explicitly recognizing community identity in terms of groups that reflect specific settings, scales, and social power structures [36–39].

#### The depth of community voice strategies

The depth of a collaborative’s community voice approach is often described using terms like “meaningful” or “authentic.” In such cases, meaningful or authentic involvement is understood as the ideal form of community voice [20, 31, 40]. These qualifiers are most often used within the context of recommendations to organizations on how to include community members in collaborative work. This language conveys the insight that community voice is often marginalized even when it is purportedly a central concern. However, as in the broader literature on community voice [18], these qualifiers often do not come with clear definitions, leaving it unclear how we might understand or identify effective practices. The qualifiers themselves are not inherently faulty, and prior research on community voice more broadly is quite clear that some community voice strategies are more likely to be emancipatory than others [41]. The point is that the frequent use of unexplained qualifiers underscores the need to identify and use measures that more specifically identify the degree to which, and how, community voice is engaged.

To summarize this section, researchers and practitioners interested in community voice are likely to gain mutual benefit from using clear definitions and qualifiers which (1) identify a precise set of community members in terms of groups that reflect specific settings, scales, and social power structures and (2) specify the type of community voice under consideration.

### Types of community voice strategies

#### Passive community voice strategies

Our review suggests that community voice strategies tend to range across two types: passive and active. Consistent with earlier research [15, 16], we found that passive forms of community voice tend to transfer less power to community members than active forms of community voice. However, we also found that passive community voice tended to place fewer resource requirements on community members on an individual basis. Passive strategies for community voice tend to take the form of collecting data from community members [42]. In the studies reviewed, three types of strategies for including passive community voice in collaborations were identified: holding community forums, measuring community intervention experiences, and conducting community assessments.

Community forums are used to share data with community members and to compare already-obtained data with community perceptions [43]. The intent is to share data and information out to community members. Community members might, for example, take part in community roundtables or working groups [44]. Because some community members may face barriers to participating including limited resources or time, there are several recommendations for making these forums more community friendly. Organizations can make meetings more accessible by holding them at convenient times; assisting community members with travel, childcare, and translation services; providing compensation for their time; and providing multiple avenues for participation, for example via the internet as well as in-person [31, 45].

A second passive community voice strategy is measuring community intervention experience. This is, in some ways, another type of community assessment; however, it focuses specifically on measuring community experience with current programming. The intent is evaluation. This may, for example, take the form of collecting customer satisfaction surveys [31, 46]. Another intervention measurement strategy is conducting formal and informal conversations with community groups to gain feedback and community perspective on programming [46–48]. Such conversations can be used to make programming more culturally appropriate [48].

The third passive community voice strategy is to conduct community assessments. Community assessments are used to measure health and well-being within a community and raise issues collaboratives might address. Assessments can take many forms but often take the form of Community Health Needs Assessments (CHNAs). The Patient Protection and Affordable Care Act (ACA) made CHNAs a requirement for tax-exempt hospitals. Part of the requirement includes participation from community members in the CHNA process. However, the wording of the requirement is vague on the definition for community participation [31]. This means that, in practice, CHNAs can be limited to community focus groups or community members helping gather data. Interaction with community members can be limited, and while this strategy places less burden on community members’ time and resources than more active strategies, it provides few opportunities for community members to make decisions about how the data is collected or used.

#### Active community voice strategies

Active community voice strategies convey more power to community members when compared with passive strategies. This power comes from having community members in decision-making roles. Active strategies position community members within collaboratives. However, while active community voice strategies often entail greater empowerment, we also found that placing community members within the collaborative came with higher capacity requirements for community members. Examples of activities requiring higher capacity include time for trainings, relationship building, and attendance at collaborative activities. Five strategies for including active community voice in collaborations were identified: priority-setting, participatory decision-making, trainings, employing community members, and community-led coalitions.

The first strategy for engaging active community voice in collaboratives is priority setting [31, 40, 42, 49]. Community members can help by identifying and collecting important data in their communities. For example, community members can work with collaboratives on designing health impact assessments (HIAs). HIAs are recognized as valuable for advocacy efforts, and communities can make them more impactful by including their voices [40]. This differs from other community assessment tools because priority setting is done in collaboration with the community. Whereas other tools, like CHNAs, in many instances can be limited to only gathering data from community members.

The second strategy is participatory decision-making. There are three forms of participatory decision-making in the literature. One form involves health consumers being active participants in their care provision and in the decision-making concerning their own care [30, 31, 33, 46, 50, 51]. Another form is community members holding seats on governing or advisory boards [31, 32, 52]. The intensity of involvement required for community members shifts between the former and the latter type of participatory decision-making. The latter type of participatory decision-making moves beyond individuals making decisions about their own care to community members making decisions that affect the care of many. This will require more of community members’ time and resources for participating in these types of activities. Examples include hospital-based patient and family advisory councils. Community involvement in these two examples typically requires at least 50 % membership by current or former patients or family members [31]. The Federal Public Health Service Act requires federally funded community health centers to have a consumer majority on their board of directors [31], and the National Multisector Health Coordinating Body has seats reserved for consumer participation [52]. A third form of participatory decision-making is participatory budgeting. Participatory budgeting empowers communities to make funding allocation decisions [31, 53]. This form of community voice gives communities power in deciding what needs to address and how those needs should be met.

Notably, complicated power dynamics are likely to remain even when community members are empowered. For example, community members can be discouraged from participation in decision-making processes by the use of technical language and jargon [27, 32]. Addressing such dynamics may require activities such as developing a shared language between collaborators and community members [20].

Another example involves power-knowledge values. The knowledge gained from research or academic conferences can be valued more than knowledge gained from protests or grassroots organizing [54]. It is important to recognize that these values impact who is allowed at the table to define community problems and solutions [54].

A third type of active community voice strategy is training. Organizations can take the time to provide training to communities, helping them develop skills that will enhance collaboration between the two entities. Trainings can, for example, address decision-making, advocacy, or how to work collaboratively [32, 40, 44]. Specialized trainings on social determinants of health can also help [40]. In many cases, community members are not the only ones needing training, and training collaborative members from across organizations can strengthen the collaborative capacity of all involved while helping to build community-collaborative relationships [40].

A fourth active community voice strategy is to hire community members into collaboratives, for example in community-liaison roles such as community health workers and community care coordinators. Such positions help connect other community members to services and have been shown to improve community health outcomes [31]. Providing monetary compensation for the additional time and expertise community members are providing can aid in their ability and motivation to participate in collaboratives [45]. Embedding community members also helps organizations and communities build trust, which has been cited many times in the literature as an important step in engaging community voice in collaborations more broadly [27, 32, 45, 48].

The fifth active community voice strategy is to create community-led coalitions. Though the papers we reviewed provided strategies for including or even embedding community voice in collaboratives, leadership originating outside the community still tends to play the dominant role in such working arrangements. However, one paper highlighted an initiative where a community coalition itself led much of the collaborative intervention [55]. In this intervention, health consumers oversaw monitoring the performance of health providers in collaboration with government. In this case, there was legislation in place that mandated citizen participation in governing health and social sectors [55]. This mandate pointed to community participation, specifically in “… planning, supervision, execution, and administration of health programs that are key actions for guaranteeing the right to health” [55]. In this initiative, community members made many of the decisions, with guidance and support provided by government collaborators. However, even with this example, program sustainability was affected by community volunteer turnover due to lack of pay for the intense level of involvement required by the initiative [55].

### Sustained systems change and community voice

One of the core purposes of the cross-sector alignment theory of change is to help guide sectors and organizations as they transition from a focus on short-term collaboration to sustained systems change. We found that the strategies identified above lend themselves to sustained change to different degrees. In this section, challenges to long-term community voice strategies are identified along with potential solutions.

#### Passive and active strategies and sustained systems change

Several of the strategies identified above are primarily oriented toward short-term community contact. These tend to be passive strategies, leading us to observe an association between sustained systems change and the intensity of community voice strategies. Specific strategies oriented toward short-term collaboration include data collection, community forums or hearings, and initiative evaluations. These activities could be institutionalized and become regular occurrences, and these strategies may be helpful in many situations. However, they tend to take the form of irregular or one-time contact, inherently limiting their long-term potential. Other strategies for engaging community voice may be more appropriate where the intent is to create sustained connections.

One short-term strategy identified above that does involve active community voice is responding to, heeding, community voice in the form of protest [54]. Protests largely originate within communities and outside collaborative-initiating organizations, but Phipps and Masuda argue that community origin alone should not disqualify protest from being considered a form of community voice [54].

Because protests come and go, the other active strategies mentioned above are perhaps more amenable to collaboration in the long-term. These involve power sharing, community participation in standing committees and boards, compensation for community participation, and community-led decision-making. All of these provide either psychological, instrumental, or financial incentives that are likely to help promote community member involvement and, ultimately, strengthen aligning efforts over the long-run.

#### Challenges and potential solutions

Challenges in implementing community voice are discussed often and may even be inherent. Several studies note that engaging communities is difficult [27, 56]. Active community voice can be difficult to retain, often requiring unexpected compromises in implementation strategies, changes in research design, shifts in priorities, delays in anticipated schedules, surrender of power to the community, and ultimately, a shift in expectations for both processes and objectives [27, 52, 57].

Many organizations are not able to, or are disinclined to, make such accommodations [27, 52, 58]. Such changes may challenge fundamental assumptions individuals have about their roles and the roles of the organizations in which they participate. Power is not merely intellectual. It is instrumental, and giving it away has material consequences. Hesitance to share power is a key factor in the devolution of many community voice activities into tokenism [41].

Not everyone is prepared to make changes in operations that alter power relations between themselves and community members [59]. However, given the emphasis in the literature on building trust [27, 60, 61], being thoughtful about boundaries is likely to encourage productive relationships even where changes in processes and outcomes are expected to be relatively moderate.

Finally, given that many of the studies we reviewed discussed the need to turn away from ineffective solutions, the need for change, and the importance of change management [62–66], sustainable collaboration may require embracing change. In terms of engaging community voice, this suggests that aligning organizations themselves should carefully consider how collaborative and community resources can be allocated in the most productive ways.

## Discussion

This review surfaced several definitions for terms linked to community and community voice. Though community voice terms tend to be used interchangeably, the literature does offer jumping-off points for the systematic use of standardized definitions. This review also underscores the need for researchers and practitioners to be explicit about their definitions of community voice and identify the community in focus in relation to specific settings, scales, and social power structures [36–39]. Furthermore, the possibility that community voice could involve different population groups implies that the term “community voice*s*” (plural) may be the more precise term in some contexts where no single community is identified [14, 67].

We also distinguished community voice strategies that varied along a continuum from passive to active. Reflecting the broader literature on community voice, these two types of strategies tend to vary in the power that is shared with communities, with more active strategies involving more power sharing. However, these strategies also tend to vary along the same spectrum in the intensity of involvement required by community members, with more empowering strategies requiring greater resources on the part of individual community members.

While prior research has identified a link between active community voice and power [20, 27, 45, 53], identified potential pitfalls with community voice strategies such as tokenism [18, 41, 52, 68], and pointed out the need for time and capacity-building when working with community partners [27, 45, 46, 52, 58, 68], no prior study of which we are aware has noted that the requirements placed on community members tend to increase *along with* power sharing on the active-passive community voice spectrum. It is critical that researchers and practitioners recognize that empowerment may pose fewer new requirements on those with resources to address the requirements of increased involvement, but empowerment in the complex environment of a collaborative may impose greater burden on people in historically under-resourced communities [68]. The burdens placed on participating community members will have to be addressed if empowerment is going to take place, and practitioners should strategically consider how those burdens may increase along with power sharing, for example by planning resources for capacity transfer along with power transfer, or by planning high-impact activities specially designed to have low burden for community members. Indeed, practitioners may find that certain activities offer higher leverage, for example in governance where community board members might be able to have a significant impact on operations and outcomes. This is of particular importance to the cross-sector alignment theory of change as it would have implications for how community voice is implemented in the development of shared purpose, data, financing, and governance, respectively. In summary, organizations should carefully consider how they facilitate more intense involvement from communities, which may require training, capacity building, time for relationship building, and fair compensation for community members’ time and efforts as community voice is brought to bear on the varied activities of a cross-sector collaborative.

Another key concern in the development of the cross-sector alignment theory of change was to promote sustainable collaboration. Active community voice strategies may be more suited to promoting sustainability since they tend to extend beyond one-time activities. However, they do pose challenges in that they require time and resources from everyone involved, and they may result in deviations from what aligning organizations or systems had originally expected in terms of processes and outcomes.

### Implementing community voice strategies

Aligning organizations may benefit greatly from community voice [30, 53, 69–71]. Community members add a sense of urgency around issues that they experience first-hand as important for their own well-being [69]. Community voice adds intellectual and experiential capacity to aligning organizations [27, 30, 70]. Community members can provide data on the community in the short term, and by incorporating community members over the long-term, for example as decision-makers and paid employees, aligning organizations can institutionalize community voice as a lasting knowledge-producing solution. Responsiveness to community voice also signifies sincere concern for community goals and needs, perhaps increasing the likelihood of financing from potential investors [72, 73].

Such advantages do not always come without costs, and those engaged in cross-sector alignment should prepare themselves – and community members – appropriately. Working with communities takes time, which can be costly and increase exposure to uncertainties. Working with communities also takes resources, both for organizations and individuals, and financing arrangements will have to be made accordingly. Governance structures may also have to change as community members are empowered in budgeting and other decision-making roles. Aligning sectors and organizations may be confronted with the need to accept the legitimacy and importance of community-led coalitions that have their own governance structures.

Despite the costs, incorporating community voice in health collaboratives appears likely to promote the objectives of cross-sector alignment, including improved community well-being. In the process, engaging with community voice may also help build capacity in communities and, ultimately, empower community members to self-advocate in pursuing positive community outcomes.

### Limitations

The studies we reviewed are primarily exploratory or descriptive in nature. Similarly, most of the studies we reviewed did not contain a theoretical analysis, limiting the linkages that could be made with research on community voice in other contexts. These limitations represent opportunities for future research. Future studies could productively incorporate strong theoretical foundations and incorporate methods that allow for causal analysis. We have attempted to move in this direction by grounding our findings in the cross-sector alignment theory of change, the literature on health collaboratives, and the broader literature on community voice. This effort will also be greatly aided where practice is based on a theoretically-informed foundation and rigorous evaluation plans are conceived and implemented early in the project planning phase.

## Conclusion

This review provides three important contributions to the field. First, we have characterized the literature on community voice in health-oriented collaborations, providing a conceptual jumping-off point for defining, discussing, and analyzing community voice as a concept in this literature. Second, we have provided a theoretical basis for future research by differentiating passive and active community voice strategies and highlighting the relationship between the power dynamics and resource requirements that undergird these strategies. Finally, we have situated the literature in respect to sustained systems change and the cross-sector alignment theory of change, thereby promoting the theoretical development of a prominent framework in the field.

There is evidence that health-oriented cross-sector collaboration can have a positive impact on community health outcomes [3–6]. This review elaborated on the differences between passive community voice strategies and active community voice strategies, which tend to provide an enhanced level of decision-making and power to community members. We expect that increased attention to the concept of active community voice will help practitioners achieve improved health outcomes and help researchers better understand the pathways to health improvement through collaboration and, ultimately, cross-sector alignment.

## Data Availability

The datasets used and/or analyzed during the current study are available from the corresponding author on reasonable request.

## Abbreviations

SDOH: Social determinants of health
RWJF: Robert Wood Johnson Foundation
CDC: Centers for Disease Control and Prevention
NASEM: National Academies of Sciences, Engineering, and Medicine
CHNA: Community Health Needs Assessment
ACA: Affordable Care Act
HIA: Health impact assessment
